# *CACNA1C* (Ca_V_1.2) and other L-type calcium channels in the pathophysiology and treatment of psychiatric disorders: Advances from functional genomics and pharmacoepidemiology

**DOI:** 10.1016/j.neuropharm.2022.109262

**Published:** 2022-09-22

**Authors:** Paul J. Harrison, Syed M. Husain, Hami Lee, Alejandro De Los Angeles, Lucy Colbourne, Arne Mould, Nicola A.L. Hall, Wilfried Haerty, Elizabeth M. Tunbridge

**Affiliations:** a Department of Psychiatry, University of Oxford, Warneford Hospital, Oxford, OX3 7JX, UK; b Oxford Health NHS Foundation Trust, Warneford Hospital, Oxford, OX3 7JX, UK; c Earlham Institute, Norwich Research Park, Norwich, NR4 7UZ, UK; d School of Biological Sciences, University of East Anglia, Norwich, UK

## Abstract

A role for voltage-gated calcium channels (VGCCs) in psychiatric disorders has long been postulated as part of a broader involvement of intracellular calcium signalling. However, the data were inconclusive and hard to interpret. We review three areas of research that have markedly advanced the field. First, there is now robust genomic evidence that common variants in VGCC subunit genes, notably *CACNA1C* which encodes the L-type calcium channel (LTCC) Ca_V_1.2 subunit, are trans-diagnostically associated with psychiatric disorders including schizophrenia and bipolar disorder. Rare variants in these genes also contribute to the risk. Second, pharmacoepidemiological evidence supports the possibility that calcium channel blockers, which target LTCCs, might have beneficial effects on the onset or course of these disorders. This is especially true for calcium channel blockers that are brain penetrant. Third, long-range sequencing is revealing the repertoire of full-length LTCC transcript isoforms. Many novel and abundant *CACNA1C* isoforms have been identified in human and mouse brain, including some which are enriched compared to heart or aorta, and predicted to encode channels with differing functional and pharmacological properties. These isoforms may contribute to the molecular mechanisms of genetic association to psychiatric disorders. They may also enable development of therapeutic agents that can preferentially target brain LTCC isoforms and be of potential value for psychiatric indications.

This article is part of the Special Issue on ‘L-type calcium channel mechanisms in neuropsychiatric disorders’.

## 1 Introduction

Several voltage-gated calcium channel (VGCC) subunit genes are found among the hundreds of loci implicating thousands of genes that have been revealed by genome-wide association studies (GWAS) of psychiatric disorders. The most notable example is *CACNA1C*, which encodes the L-type calcium channel (LTCC) Ca_V_1.2 pore-forming subunit.

In many respects the GWAS signals for VGCCs are no more impressive nor transformative than most others: the effect sizes are trivial, and the causal polymorphisms, molecular mechanisms, and functional correlates of the genetic variation are unknown. However, viewed in the wider context, the demonstrated involvement of VGCC genes does have greater implications. Firstly, LTCCs and other VGCCs play a well-established role in fundamental neuronal processes relevant to psychiatric disorders, including transmitter release, synaptic plasticity, and excitation-transcription coupling (Striessnig et al., 2014; Zamponi et al., 2015; Nanou and Catterall, 2018) and impact on learning, memory, and other relevant behaviours (Wankerl et al., 2010; Kabir et al., 2017). Secondly, complementing the GWAS data, there is evidence for rare VGCC variants with penetrant effects on disease risk. Thirdly, altered intracellular calcium signalling has been documented in cells from patients with psychiatric disorders, especially bipolar disorder and depression (Dubovsky and Franks, 1983; Berridge, 2014; Harrison et al., 2021b). Complemented by emerging understanding of the structure of VGCCs (Wu et al., 2015; Tang et al., 2016), these considerations together provide a strong biological framework within which to interpret the genomic evidence. Finally, and significantly, VGCCs are druggable: calcium channel blockers (CCBs), which target the α1 subunit of LTCCs (Striessnig et al., 1998), are widely used for hypertension and other cardiovascular indications (Braunwald, 1982), whilst the gabapentinoids, which act via the α2δ subunits (Gee et al., 1996), are used for several conditions including sleep disorders, epilepsy and pain (Hong et al., 2022).

Here we summarise: (a) genomic data implicating LTCCs in psychiatric disorders; (b) epidemiological evidence for beneficial therapeutic effects of CCBs in these disorders; and (c) molecular studies revealing novel LTCC isoforms that may mediate the genetic associations and provide genomically-informed psychotropic drug targets. As outlined below, LTCCs are likely of relevance to brain processes that cut across psychiatric diagnostic boundaries. However, given that the genomic evidence has been collected using current diagnostic criteria and that the most robust associations are seen for *CACNA1C*, we focus here on *CACNA1C*’s involvement in psychosis (schizophrenia and bipolar disorder). Nevertheless, many of the issues pertain to other LTCC genes and other neuropsychiatric disease phenotypes (Heyes et al., 2015; Zamponi, 2016).

## 2 LTCC genes contribute to risk for many psychiatric disorders

The first genome-wide significant association between an LTCC gene and a psychiatric disorder was for *CACNA1C* and bipolar disorder (Sklar et al., 2008; Ferreira et al., 2008). This was followed by a number of associations between this and other VGCC loci with several psychiatric disorders, especially schizophrenia, as well as with a cross-disorder phenotype (Table 1; Cross-Disorder Group of the Psychiatric Genomics Consortium, 2013; Bipolar Disorder and Schizophrenia Working Group of the Psychiatric Genomics Consortium, 2018; Mullins et al., 2021; Trubetskoy et al., 2022). The latest GWAS results for schizophrenia now include significant associations to three of the four LTCC α1 subunit gene loci (Trubetskoy et al., 2022).

The cumulative GWAS data strongly suggest a role for common variation in VGCC subunit genes, especially *CACNA1C*, in the genetic architecture of severe mental illness (Casamassima et al., 2010; Bhat et al., 2012; Harrison et al., 2021b). However, as with all GWAS findings, many questions remain to be addressed, beyond the basic limitation that the causal gene(s) and variant(s) at each locus need to be determined (Harrison, 2015; Wang et al., 2019; Mountjoy et al., 2021; Wainberg et al., 2022). Firstly, what is the molecular mechanism of the genetic association? The genomic signals are non-coding, and so likely impact on gene regulation and expression. However, studies to date are inconclusive as to the direction of effect, if any, of the risk alleles on *CACNA1C* mRNA abundance (Bigos et al., 2010; Gershon et al., 2014; Yoshimizu et al., 2015; Jaffe et al., 2020); the issue is relevant since altered *Cacna1c* expression in rodents affects their behavioural phenotype (see Moon et al., 2018). The variable results may reflect temporal or spatial variation of the allelic effect, for example across neurodevelopment or between brain regions or cell types. Alternatively, given that the GWAS signal arises from an intron within the gene, a plausible mechanism of association is that it involves altered splicing and thence production of specific isoforms rather than an overall increase or decrease in *CACNA1C* expression. This possibility has yet to be tested, but a genotype effect on splicing is a key event for other psychosis risk genes (Kleinman et al., 2011; Tao et al., 2014; Xiao et al., 2017; Gandal et al., 2018; Zhang et al., 2022). Secondly, what is the downstream effect of the disease-associated genetic variation on the properties of the encoded channel and thence the processes and networks in which they participate? There are some findings showing *CACNA1C* genotype influences channel characteristics (Yoshimizu et al., 2015; Birey et al., 2017), and similarly for *CACNA1I* (Baez-Nietro et al., 2022), but the overall picture remains unclear, in part because the existence of any genotype-associated isoform(s) has yet to be shown. It is also unclear how the LTCC findings relate to the dysregulation of intracellular calcium signalling observed in several psychiatric disorders noted above, especially since much of that work was carried out in platelets and lymphocytes. However, the increasing evidence for expression and function of LTCCs in non-excitable cells indicates that a link of some kind is possible (Alves et al., 2019; Pitt et al., 2021). Thirdly, the pleiotropic nature of genetic associations across disorders raises the question as to which aspects of the clinical phenotype, as well as the pathophysiology, the LTCCs participate in (Lee et al., 2021). A parsimonious explanation is that they contribute to the processes underlying one or more of the many and diverse features observed *trans*--diagnostically across the disorders, such as cognitive impairment, mood instability, sleep difficulties, or physical comorbidity. The temporal profile of LTCC expression in brain across development also needs to be borne in mind when considering how, when, and where the genes exert their influence (Dedic et al., 2018; Clifton et al., 2021; Hall and Bray, 2022). Clarifying these various genotype-phenotype relationships will be one of the issues to be resolved before the role of LTCCs in disease pathogenesis can be understood, and any potential of LTCCs as psychotropic drug targets can be realized.

Complementing the common variation, rare variants in some LTCCs, and other VGCCs, also confer risk for psychiatric disorders and neurodevelopmental syndromes (Table 1). The paradigm example is Timothy syndrome, in which autistic features are prominent together with a cardiac and skeletal phenotype, caused by a gain-of-function mutation in *CACNA1C* (Splawski et al., 2004) which acts, at least in part, by altering splicing of the gene (Panagiotakos et al., 2019). Rare (Purcell et al., 2014; Wang et al., 2022) and structural (Song et al., 2018) variants in *CACNA1C* have also been reported in psychosis, and rare variants in *CACNA1C* and *CACNA1D* identified in other neurodevelopmental syndromes (Pinggera et al., 2015; Ortner et al., 2020; Rodan et al., 2021). Although of limited population impact, rare variants are invaluable because they provide greater traction on the underlying biology, and the findings may give clues as to the mechanisms underlying the common variant associations.

In summary, there is now strong evidence that LTCCs are part of the genetic architecture of a range of psychiatric disorders. Attention can now turn to understanding the nature and mechanisms of the LTCC contribution to aetiology, and whether and how this understanding can be harnessed to advance the candidacy of these channels as targets to treat psychiatric disorders.

## 3 Calcium channels and blockers in psychiatry

Initial interest in the possible role of LTCCs in the treatment of psychiatric disorders was stimulated by the introduction of the first calcium channel blockers (CCBs), verapamil and diltiazem, for treatment of hypertension and other cardiovascular indications. This chimed with a hypothesized role of aberrant calcium signalling in bipolar disorder and depression that was emerging around that time (Crammer, 1977; Jimerson et al., 1979; Dubovsky and Franks, 1983; Bowden et al., 1988), and with the fact that some antipsychotic drugs were shown to be calcium channel antagonists, potentially contributing to their mode of action (Gould et al., 1983). A number of case reports, case series, and small clinical trials followed, in which patients with bipolar disorder, depression or schizophrenia were treated with CCBs, mainly verapamil. Despite initial enthusiasm, findings were not robust and interest waned (Hollister and Trevino, 1999), and a systematic review in bipolar disorder concluded that there was an absence of evidence from randomized controlled trials to support their use (Cipriani et al., 2016).

Discovery that LTCCs are part of the genetic risk architecture for neuropsychiatric disorders has rekindled interest in these channels as therapeutic targets (Dubovsky, 2019; Harrison et al., 2020b). Contemporary therapeutic investigations are using the newer dihydropyridine CCBs (Ostacher et al., 2014; Atkinson et al., 2019; Burdick et al., 2020; Vahdani et al., 2020). For example, Ostacher et al. (2014) studied effects of isradipine on bipolar disorder, selecting patients based on *CACNA1C* risk genotype. However, these recent studies are all exploratory or pilot in nature, and have not yielded any conclusive results. While larger clinical trials are awaited, two further approaches are being adopted to advance the candidacy of CCBs in psychiatry: the first is to use pharmacoepidemiology to provide clues as to whether the existing CCBs are associated with differences in risk for, or outcome of, psychiatric disorders. The second approach is to explore the identity and characteristics of LTCCs and their subunits in more detail, with particular regard to the possibility that they may differ in the brain compared to peripheral tissues. These issues are relevant both for explaining the mechanisms by which the genes may contribute to risk for psychiatric disorders, and for the possibility that modified CCBs – or other therapeutic agents interacting with these channels in order to enhance, inhibit, or modulate their functioning – could be of value.

### 3.1 Pharmacoepidemiology

The advent of electronic health records has enabled large-scale pharmacoepidemiological studies of patients who are prescribed CCBs for cardiovascular indications to study whether their use is associated with an altered incidence or course of psychiatric disorders. A study of the Swedish population found that patients with schizophrenia or bipolar disorder had fewer psychiatric hospital admissions when they were taking CCBs compared to when they were not (Hayes et al., 2019). Other studies have compared people taking CCBs with people taking another class of antihypertensive drug, to control for the confounding effect of hypertension. Being conducted more recently than the initial clinical studies mentioned above, the pharmacoepidemiological work focuses on the dihydropyridine CCBs which have largely superseded use of verapamil and diltiazem; of the dihydropyridines, amlodipine is much the most frequently prescribed.

The pharmacoepidemiological findings are somewhat varied, but overall there is evidence for a reduced risk of onset or recurrence of depression with CCBs compared to beta-blockers, and some evidence for CCBs compared to diuretics; in contrast, CCBs are inferior compared to angiotensin II receptor blockers (Boal et al., 2016; Cao et al., 2019; Kessing et al., 2019; Agustini et al., 2020; Colbourne et al., 2021; Shaw et al., 2021). A broadly similar pattern for CCBs relative to other anti-hypertensive classes pertains for several other psychiatric disorders (Colbourne et al., 2021), delirium (Harrison et al., 2020a), and neurodegenerative disorders (see Harrison et al., 2021a).

**Fig. 1:**
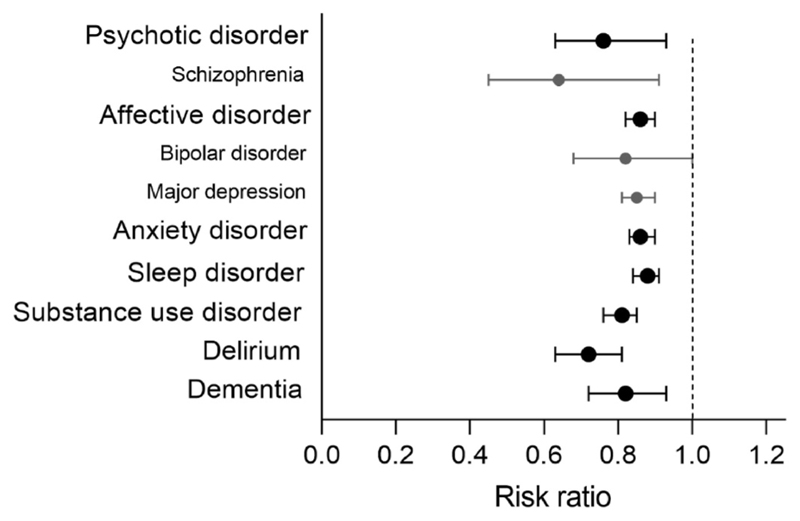
Brain-penetrant calcium channel blockers and risk of psychiatric disorders. The incidence of a first recorded psychiatric diagnosis during a two-year period is lower in patients taking a brain-penetrant CCB than those taking amlodipine, a CCB with low brain permeability (n = 44,732 in each cohort). Cohorts were propensity score matched at baseline for age, sex, race, blood pressure, body mass index, and for a range of medical diagnoses and medications. Risk ratios are shown with 95% confidence intervals. Similar results were seen when brain-penetrant CCBs were compared with verapamil and diltiazem. Data taken from Colbourne and Harrison (2022).

An important caveat is that these pharmacoepidemiological studies considered CCBs as a class, and did not take the varying blood-brain barrier penetrability of individual drugs into account. It seems likely that any psychiatric benefits of CCBs would be greater for those drugs that do enter the brain and thereby have the opportunity to block neuronal LTCCs compared to those that do not. Notably, three of the CCBs mentioned thus far – verapamil, diltiazem, and amlodipine – all have low blood-brain barrier penetrability unlike the other dihydropyridines (such as felodipine, isradipine and nifedipine). We tested this hypothesis in a retrospective cohort study using a large electronic health records network (Colbourne and Harrison, 2022). We found that, over a two-year exposure period, the brain-penetrant drugs were associated with a lower incidence of a first psychiatric diagnosis compared to the other CCBs. This applied across a range of common disorders, as well as for delirium and dementia, with an overall risk reduction of 12% (Fig. 1). Small reductions in risk were also seen for recurrence of prior diagnoses. In addition, the brain-penetrant CCBs were associated with a lower overall incidence of psychiatric disorders compared to the angiotensin receptor blockers. This in contrast to the earlier finding that angiotensin II receptor blockers were associated with a lower incidence when compared to *all* CCBs (of which the large majority of prescriptions were for non-penetrant CCBs).

Pharmacoepidemiological studies are observational, subject to residual confounding, and cannot demonstrate causality nor mechanism. Nevertheless, the results suggest that brain-penetrant CCBs may have some relative benefits on the risk of common psychiatric disorders. As such the findings provide impetus to explore further the role of brain LTCCs in these disorders and as treatment targets for novel psychotropic drugs. Notwithstanding this rationale, the existing CCBs, even those that readily access the brain, are likely not optimal at occupying central LTCCs, and even if this could be achieved at higher doses, it would come at the price of significant side-effects due to their cardiovascular actions. A more attractive option would be to modify CCBs to enable them to preferentially block brain VGCCs.

### 3.2 Towards brain-selective LTCC drugs

The potential to selectively target brain LTCCs is afforded by the presence of brain-enriched isoforms. (Note that the term ‘isoform’ is sometimes used to refer to LTCC subtypes, e.g. Ca_V_1.2 vs Ca_V_1.3, but here we use it in its formal sense to refer to alternative mRNA transcripts and proteins expressed from a single gene). It has been known for a while that alternative splicing of specific exons within individual LTCC genes is an important mechanism contributing to the functional diversity of the resulting channels (Lipscombe et al., 2013; Lipscombe and Andrade, 2015). *CACNA1C* contains many alternatively spliced exons, including exons 8/8A, involved in Timothy syndrome, and exons 21/22, 31/31a, and 33. These exons have been shown in various ways to impact upon channel kinetics, state-dependent inactivation, and dihydropyridine binding (Soldatov et al., 1995; Zuhlke et al., 1998; Liao et al., 2007; Tiwari et al., 2006; Hu et al., 2017; Li et al., 2017). These splicing events can be developmentally regulated and tissue-specific, including some exons that are retained in brain (e.g. exon 22; Tang et al., 2007).

Recently, application of long-range cDNA sequencing has revealed many new splicing events in LTCCs and enabled identification of the repertoire of full-length transcripts within which alternative exons occur (Clark et al., 2020). Prior to these technical advances, methods primarily focused on individual exons or segments of the gene, and could not readily detect previously unannotated exons. The long-read data highlight that pre-existing transcriptome annotations for *CACNA1C* are far from complete, and give a misleading picture (Clark et al., 2020; Hall et al., 2021). Importantly, several of the newly identified human *CAC-NA1C* isoforms are more abundant than the annotated isoforms, and are enriched in brain compared to heart or aorta (NALH, SMH, HL, PJH, WH, EMT, unpublished observations). Moreover, some of the new isoforms are predicted to encode channels that vary in their function and/or pharmacology. The isoforms arise from multiple sources, including alternative transcription start sites, inclusion of novel exons, and different combinations of known exons. There is also heterogeneity at the 3^′^-end encoding the carboxy terminal domain. Some of the novel isoforms are common to humans and mice, whereas others differ between these species (SMH, NALH, PJH, WH, EMT, unpublished observations).

Many questions remain regarding the significance of these observations. It is not known which of the novel transcripts are translated and whether transcript abundance predicts protein abundance. Nor is it known where they are expressed (in terms of cell type and subcellular location), nor how they are regulated. It also remains to be determined whether the novel isoforms contribute to the mechanism by which genetic variants in *CACNA1C* influence risk for psychiatric disorders. Furthermore, the predicted functional and pharmacological differences between the predominant isoforms in brain and periphery need to be empirically tested using a range of methods. Given the species differences mentioned, the approaches will need to include human model systems, such as induced pluripotent stem cell-derived neurons and other relevant cell types (De Los Angeles et al., 2021). Finally, it is important to bear in mind that even an optimal centrally-acting dihydropyridine may not achieve full blockade due to the membrane potential of neurons (Xu and Lipscombe, 2001).

Notwithstanding these important caveats, the recent molecular findings provide a tantalizing possibility that brain-enriched isoforms of *CACNA1C* - and other VGCCs - could be preferentially targeted, allowing therapeutic agents for psychiatric disorders with greater central potency and fewer peripheral side-effects (Harrison et al., 2020b; Hall and Tunbridge, 2021). Certainly, their existence complements the opportunities, and difficulties, that exist in terms of identifying drugs that can target one LTCC selectively from another, such as Ca_V_1.2 vs. Ca_V_1.3 (Zamponi et al., 2015; Ortner et al., 2017; Wang et al., 2018; Lanzetti and Di Biase, 2022).

## 4 Conclusions

The diverse and cumulatively compelling evidence outlined above suggests that LTCCs, and some other VGCCs, contribute to the mechanisms underlying serious mental illnesses. Compared to most of the genes linked to these disorders, considerably more is known about the function and structure of the encoded proteins. Critically, CCBs and gabapentinoids show that LTCCs are druggable and, moreover, that these drugs can impact on psychiatric phenotypes. These factors together give LTCCs a high priority in the efforts to leverage genomic discoveries to clarify the pathophysiology and advance the pharmacotherapy of serious psychiatric disorders (Breen et al., 2016; Birnbaum and Weinberger, 2020; Harrison et al., 2022). Detailed functional characterization of LTCC gene isoforms that are preferentially expressed in brain, and those that are modulated by psychiatric risk genotype, will be a key part of this endeavour.

## Figures and Tables

**Table 1:** Common and rare variant associations of LTCC and other VGCC genes with psychiatric disorders.

Subunit type	Channel type^a^	Channel name^a^	Subunit name	Gene symbol	Common variants^b,c^	Rare variants^b,d^
Alpha1 (α1)	L-type	Ca_v_1.1	α1S	*CACNA1S*	**Scz**, XD	Scz
		Ca_v_1.2	α1C	*CACNA1C*	**BD**, **Scz**, ASD, XD	ASD, BD, Scz
		Ca_v_1.3	α1D	*CACNA1D*	**Scz**, XD	ASD, BD
		C3_v_1.4	α1F	*CACNA1F*		
	P/Q-type	Ca_v_2.1	α1A	*CACNA1A*		
	N-type	Ca_v_2.2	α1B	*CACNA1B*	**BD**	BD, Scz
	R-type	Ca_v_2.3	α1E	*CACNA1E*	MDD, XD	
	T-type	Ca_v_3.1	α1G	*CACNA1G*		**Scz**
		Ca_v_3.2	α1H	*CACNA1H*		ASD, Scz
		Ca_v_3.3	α1I	*CACNA1I*	**Scz**, ASD	
Beta (β)			β1	*CACNB1*		
			β2	*CACNB2*	**Scz**, **BD**, XD	ASD
			β3	*CACNB3*		
			β4	*CACNB4*		Scz
Alpha2delta (α2δ)			α2δ1	*CACNA2D1*	MDD	Scz
			α2δ2	*CACNA2D2*	**Scz**, XD	Scz
			α2δ3	*CACNA2D3*		ASD
			α2δ4	*CACNA2D4*	XD	Scz

a Channel type and name are defined by the α1 subunit.

b ADHD: attention-deficity hyperactivity disorder; ASD: autistic spectrum disorder. BD: bipolar disorder. MDD: major depression. Scz: schizophrenia. XD: cross-disorder (scz/BD/MDD/ADHD/ASD).

c Common variant locus associations reported in one or more GWAS. Results in boldface are genome-wide significant in the latest Psychiatric Genomics Consortium analyses of schizophrenia (Trubetskoy et al., 2022) and bipolar disorder (Mullins et al., 2021).

d Boldface denotes significant findings in the SCHEMA whole exome study of schizophrenia (Singh et al., 2022).

## Data Availability

No data was used for the research described in the article.
